# Altered Evening Aperiodic Activity and Microstate Dynamics in Insomnia Disorder: An OPM MEG Study

**DOI:** 10.1002/cns.71066

**Published:** 2026-07-30

**Authors:** Feng Cui, Kejia Hu, Hui Zeng, Yang Wang, Xi Wang, Haiting Liu, Tangfen Wang, Yan Chang, Xiaodong Yang, Tao Hu, Tianxiao Shen, Jianbing Zhu, Jian Zheng, Donghua Pan

**Affiliations:** ^1^ Suzhou Research Center of Medical School, Suzhou Hospital, Affiliated Hospital of Medical School Nanjing University Suzhou China; ^2^ Suzhou Hospital, Affiliated Hospital of Medical School Nanjing University Suzhou China; ^3^ School of Biomedical Engineering (Suzhou), Division of Life Science and Medicine University of Science and Technology of China Hefei China; ^4^ Suzhou Institute of Biomedical Engineering and Technology Chinese Academy of Sciences Suzhou China; ^5^ Department of Neurosurgery, Center for Functional Neurosurgery, Ruijin Hospital Shanghai Jiao Tong University School of Medicine Shanghai China; ^6^ Emergency & Critical Care Medicine Center, Ruijin Hospital Shanghai Jiao Tong University School of Medicine Shanghai China; ^7^ Shanghai Institute of Aviation Medicine, Ruijin Hospital Shanghai Jiao Tong University School of Medicine Shanghai China; ^8^ Department of Respiratory Medicine, Suzhou Hospital, Affiliated Hospital of Medical School Nanjing University Suzhou China; ^9^ Tongan Branch, Suzhou Hospital, Affiliated Hospital of Medical School Nanjing University Suzhou China; ^10^ Suzhou Metanetic Co, Ltd Suzhou China; ^11^ Department of Neurosurgery The First Affiliated Hospital of Wannan Medical College (Yijishan Hospital of Wannan Medical College) Wuhu China; ^12^ School of Electrical Engineering and Automation Harbin Institute of Technology Harbin China; ^13^ Zhengzhou Research Institute Harbin Institute of Technology Zhengzhou China

**Keywords:** aperiodic activity, cortical excitability, insomnia disorder, microstates, OPM‐MEG

## Abstract

**Background:**

Insomnia disorder (ID) is characterized by hyperarousal, yet the relationship between cortical excitability and large‐scale network dynamics remains incompletely understood. While fMRI studies indicate network alterations in ID, high‐temporal‐resolution characterization of these dynamics across the sleep–wake cycle is lacking.

**Methods:**

Twenty‐six patients with ID and 29 healthy controls underwent eyes‐closed resting‐state OPM‐MEG recordings during evening (pre‐sleep) and morning (post‐awakening) sessions. We analyzed the aperiodic spectral exponent to index cortical excitability and employed microstate analysis to quantify fast network dynamics. A mediation analysis was conducted to explore the associations between electrophysiological features and sleep quality.

**Results:**

Compared to controls, patients with ID exhibited a significantly flatter aperiodic power spectrum in the evening, suggesting elevated cortical excitability. Microstate analysis revealed distinct spatiotemporal alterations: (1) an evening‐specific increase in the coverage of a putative temporal‐limbic microstate, and (2) a sustained elevation of a putative sensorimotor microstate observed in both evening and morning sessions. Mediation analysis indicated that the altered evening limbic microstate dynamics statistically mediated the association between the aperiodic exponent and subjective sleep disturbance measures.

**Conclusions:**

These findings indicate that ID involves concurrent disruptions in aperiodic neural activity and microstate temporal organization. The study highlights distinct diurnal profiles for putative sensorimotor and limbic network alterations, suggesting that OPM‐MEG can effectively capture the multifaceted electrophysiological signatures of the insomnia phenotype.

## Graphical abstract

Using wearable OPM‐MEG, this study characterized evening and morning resting state neurophysiology in insomnia disorder. A flatter evening aperiodic spectral exponent suggested relatively elevated cortical excitability, alongside altered microstate dynamics involving putative limbic and sensorimotor network states. Exploratory mediation analysis further suggested that evening microstate alterations may statistically link aperiodic neural activity with subjective sleep disturbance severity, as indexed by PSQI, without implying causal inference.

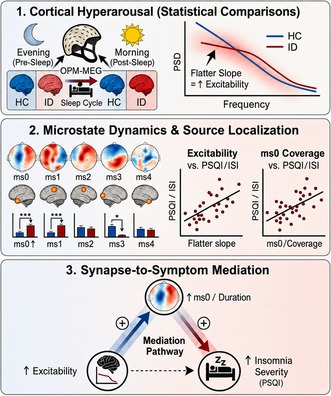

## 1 Introduction

Insomnia disorder (ID) constitutes a major public health concern, with recent systematic analyses estimating a global prevalence of approximately 16.2% among adults [1, 2]. Beyond reduced sleep duration, ID is increasingly conceptualized as a condition of persistent 24‐h hyperarousal, characterized by sustained activation of cortical, autonomic, and affective systems across the sleep–wake cycle [2, 3]. Longitudinal evidence indicates that chronic ID confers elevated risk for mood disorders and is associated with impairments in attention and executive control [4, 5]. Despite these well‐documented consequences, clinical diagnosis and treatment stratification remain largely dependent on subjective reports, underscoring the need for objective neurophysiological biomarkers that capture the underlying pathophysiology of the disorder [6].

Efforts to characterize the neural substrates of ID‐related hyperarousal have primarily relied on structural and functional magnetic resonance imaging (fMRI), revealing widespread alterations within salience, emotion regulation, and default mode networks [7, 8, 9]. Recently, dynamic fMRI modeling has significantly advanced this understanding; notably, Wei et al. demonstrated that patients with ID exhibit a rigidity in brain state transitions which correlates with symptom severity [10]. However, while this highlights the importance of network dynamics, fMRI inherently reflects slow hemodynamic processes that lag neural activity by several seconds. This limits its ability to resolve the rapid, sub‐second reconfigurations of large‐scale brain networks [11]. Such fast‐evolving dynamics are increasingly recognized as fundamental for cognitive integration and for transitions between vigilance states, including the progression from wakefulness to sleep [12]. To bridge the gap between the hemodynamic rigidity observed in recent fMRI studies and its electrophysiological origins, methods with temporal precision matched to neural processing timescales are required.

Microstate analysis of electroencephalography (EEG) and magnetoencephalography (MEG) provides a principled framework for addressing this limitation. By segmenting spontaneous neural activity into sequences of quasi‐stable topographies lasting approximately 59 to 120 ms, microstates index transient configurations of large‐scale functional networks that correspond to canonical resting‐state systems observed with hemodynamic imaging [13, 14, 15, 16]. In healthy individuals, microstate dynamics systematically vary across vigilance states and reflect sleep homeostatic regulation [17, 18, 19]. Altered microstate syntax has been established as a candidate endophenotype in several psychiatric disorders, including schizophrenia and affective conditions [20, 21]. In contrast, the application of microstate analysis to ID remains limited. Existing studies have largely focused on single time‐point recordings [22, 23], providing an incomplete view of the temporal evolution of network dynamics across the sleep–wake cycle.

Temporal switching of network states represents only one dimension of brain physiology relevant to ID. Recent methodological advances have highlighted the biological significance of the aperiodic component of the neural power spectrum, previously treated as background noise [24]. The spectral exponent has been associated with population‐level cortical excitability and arousal‐related neural dynamics, although its physiological interpretation remains indirect and context‐dependent. Accumulating evidence links aperiodic activity to arousal regulation and attentional states, suggesting that it may capture a complementary physiological aspect of ID‐related hyperarousal. Jointly characterizing microstate dynamics and aperiodic spectral properties therefore offers an opportunity to integrate temporal network organization with global cortical excitability. To date, this combined approach has rarely been applied in clinical sleep research. Crucially, no study has yet interrogated how the coupling between aperiodic activity and microstate syntax evolves across the evening‐to‐morning transition—a critical window for understanding the potential failure of homeostatic recovery in ID.

In parallel, accurate spatial characterization of electrophysiological patterns remains essential for mechanistic interpretation. While the majority of microstate studies rely on scalp EEG, MEG offers several advantages, including reference‐free recording and reduced signal distortion due to volume conduction [13, 25, 26]. The development of optically pumped magnetometer‐based MEG (OPM‐MEG) has further expanded these capabilities. Wearable OPM sensors are positioned directly on the scalp, substantially improving signal‐to‐noise ratio while allowing natural head movement, features particularly suitable for restless or clinical populations [27, 28, 29, 30]. However, OPM‐MEG has not yet been leveraged to examine the circadian evolution of microstate dynamics and aperiodic activity in ID.

In the present study, we specifically test the hypothesis that insomnia is associated with a failure to reduce cortical excitability prior to sleep, which may be accompanied by altered stabilization of brain networks in maladaptive states. Using wearable OPM‐MEG, we tracked the evolution of aperiodic activity and microstate dynamics from evening to morning. We predicted that evening cortical hyperarousal—indexed by a flatter spectral slope—would be associated with the abnormal expansion of limbic‐associated microstates. Furthermore, we postulated that this rigidity in network dynamics may statistically link physiological arousal to the subjective experience of sleep severity.

## 2 Methods

### 2.1 Participants

Fifty‐five adults (26 insomnia disorder [ID], 29 healthy controls [HC]) were recruited between September 2024 and August 2025 at Suzhou Hospital, Nanjing University Medical School. ID diagnosis followed DSM‐5 criteria: Pittsburgh Sleep Quality Index (PSQI) ≥ 8 and Insomnia Severity Index (ISI) ≥ 11 [31, 32]. HCs met criteria for good sleep (PSQI ≤ 7, ISI ≤ 10) and regular sleep schedules. Although these screening thresholds were set to exclude clinical‐level sleep disturbances rather than to select optimal sleepers, the actual recruited HC cohort exhibited excellent sleep quality well below these limits (mean PSQI: 3.66 ± 1.17; mean ISI: 3.79 ± 1.66). Exclusion criteria included shift work history, other sleep disorders (screened via clinical interview), neurological or psychiatric conditions, and current use of psychotropic medications (including melatonin). Participants agreed to abstain from caffeine, alcohol, and nicotine for at least 24 h prior to each scanning session.

### 2.2 Clinical Assessment

Demographic information (age, sex, body mass index [BMI], education) was collected at enrollment. Global cognitive function was assessed using the Chinese versions of the Mini‐Mental State Examination (MMSE) and Montreal Cognitive Assessment (MoCA). To ensure normal cognitive status, scores on either scale indicating clinically significant cognitive impairment served as an exclusion criterion. All participants completed a 7‐day sleep diary prior to the scans to verify habitual sleep patterns [33].

### 2.3 Experimental Procedure

Each participant completed two OPM‐MEG sessions within a single sleep–wake cycle: An evening session scheduled 90 ± 15 min before their habitual bedtime, and a morning session conducted 90 ± 15 min after awakening. Bedtime and wake times were verified via sleep diaries and smartwatch monitoring. Participants arrived at the laboratory 1 h before the evening session to acclimatize (< 50 lx ambient lighting). Between sessions, participants returned home to sleep to preserve ecological validity. Research staff ensured protocol compliance via text or telephone contact. During data acquisition, participants sat upright in a magnetically shielded room (MSR) with their eyes closed and were instructed to relax without performing structured cognitive tasks. Continuous video monitoring and post hoc verbal queries confirmed that participants remained awake throughout the recording. Each session comprised 10 min of resting‐state recording, divided into two 5‐min blocks separated by a 30‐s eyes‐open break.

#### 2.3.1 Sleep Verification

To objectively monitor sleep–wake schedules and verify protocol compliance, participants wore a consumer‐grade smartwatch (Apple Watch Series 9; Apple Inc., Cupertino, CA) throughout the study period. While polysomnography remains the gold standard, validation studies indicate that Apple Watch algorithms exhibit moderate‐to‐high concordance with actigraphy and polysomnography for estimating sleep parameters in naturalistic settings [34, 35]. Sleep metrics were derived using the device's integrated sleep tracking algorithms. Intraclass Correlation Coefficients (ICCs) were computed to assess the consistency between subjective (sleep diary) and objective (smartwatch) measures using a two‐way mixed model with absolute agreement.

### 2.4 OPM‐MEG Acquisition

Neuromagnetic signals were acquired using a 64‐channel optically pumped magnetometer‐based MEG system (Metalia OPM‐MEG, Suzhou Metanetic Co. Ltd., Suzhou, Jiangsu, China). Data collection was conducted inside a compact magnetically shielded room measuring 2.2 m × 1.7 m, which was equipped with active shielding coils to dynamically suppress residual ambient magnetic fields and reduce the risk of sensor saturation. The system consisted of the magnetically shielded enclosure, the OPM‐MEG acquisition unit, and an ergonomically designed seating apparatus.

Within the shielded environment, the residual magnetic field around the participant's head was approximately 2 nT, with a corresponding magnetic field gradient of approximately 3 nT per meter. The OPM sensors exhibited a noise floor of approximately 15 fT per square root hertz and a bandwidth exceeding 250 Hz.

As individual structural MRI data were not available, scalp surface geometry was acquired prior to each recording session using a handheld optical 3D scanner (EinScan H2, Shining 3D Technology Co. Ltd., Hangzhou, China). The digitized head surface was subsequently co‐registered to the MNI152 aligned fsaverage template using a rigid body transformation based on scalp shape features. Neuromagnetic signals were sampled at 1,000 Hz, with integrated real‐time optical head motion tracking applied throughout the recording session.

### 2.5 Preprocessing

Data preprocessing was performed using MNE‐Python [36]. Raw OPM signals were band‐pass filtered between 3.5 and 40 Hz using a zero‐phase FIR filter. The high‐pass cutoff of 3.5 Hz was selected to attenuate low‐frequency drift inherent to OPM sensors while preserving theta band activity; the low‐pass cutoff of 40 Hz was applied to focus on relevant resting‐state oscillations. Bad channels flagged during acquisition were inspected and interpolated. Independent Component Analysis (ICA) was performed (Picard algorithm) to identify and remove physiological artifacts, specifically cardiac (magnetocardiogram [MCG]) and ocular (electrooculogram [EOG]) components. Segments containing gross motion artifacts exceeding 5 mm were excluded prior to metric estimation.

### 2.6 Microstate Analysis and Source Reconstruction

Microstate analysis was adapted for MEG sensor‐space data following established pipelines [13, 37]. Global Field Power (GFP) was computed at each time point, and topographies at GFP peaks were extracted for clustering. A global joint clustering was performed: GFP‐peak topographies from all participants (both ID and HC) and all sessions (both evening and morning) were pooled together and submitted to a polarity‐invariant modified k‐means algorithm (5,000 random initializations). This global template approach is methodologically required to ensure that subsequent statistical comparisons of temporal parameters across groups and time points are based on an identical spatial framework. The number of microstate classes was selected using the Kneedle elbow criterion applied to the Global Explained Variance (*GEV*) curve. Evaluating model orders from *K* = 3 to *K* = 7 yielded *GEV*s of 54.70%, 61.35%, 65.73%, 68.36%, and 71.54%, respectively. The marginal gain in GEV diminished markedly after *K* = 5 (+4.38% for *K* = 4 → 5 vs. +2.63% for *K* = 5 → 6), indicating that five classes provided a reasonable balance between model complexity and explanatory power (*GEV* = 65.73%). The resulting five group‐level template maps were then back‐fitted to each individual's continuous data: At every time point, the sensor topography was spatially correlated (absolute value, to account for polarity ambiguity) with each of the five template maps, and the microstate class yielding the highest correlation was assigned. A sliding‐window mode filter (window = 30 samples, 30 ms at 1,000 Hz sampling rate) was subsequently applied to suppress spurious rapid‐state switches. From the resulting microstate sequences, temporal parameters were extracted: mean duration (ms), occurrence (times/s), and fractional coverage (%). Transition probabilities were estimated by counting frame‐by‐frame state changes across the entire sequence and row‐normalizing the resulting count matrix into a first‐order Markov transition probability matrix. To facilitate neuroanatomical interpretation despite the absence of individual MRI, a template‐based source reconstruction was employed. Individual digitized head shapes were rigidly co‐registered to the fsaverage template MRI, isotropically scaled to match head size. A forward model was constructed using a single‐shell approximation. Depth‐weighted linearly constrained minimum variance (LCMV) beamformers were computed from sensor covariance matrices. Sensor‐level microstate topographies were projected into source space, and source estimates were averaged within Desikan–Killiany atlas parcels. Given the absence of individual structural MRIs and the reliance on template‐based reconstruction, these source‐level anatomical labels should be regarded as approximate and exploratory rather than precise localizations.

### 2.7 Aperiodic and Periodic Component Analysis

Sensor‐level Power Spectral Densities (PSD) were computed using Welch's method (2‐s Hanning windows, 50% overlap). Values were converted to fT^2^/Hz and log‐transformed (dB) to normalize the data distribution. Channel‐wise PSDs were parameterized independently using the FOOOF algorithm [24] to decompose the neural power spectrum into aperiodic and periodic components. FOOOF was configured with the following settings: Frequency range = 3.5–40 Hz, peak width limits = [1.0, 8.0] Hz, maximum number of peaks = 6, minimum peak height = 0.05, peak threshold = 2.0 standard deviations, and aperiodic mode = ‘fixed’ (appropriate for frequency ranges below 100 Hz). Under the fixed aperiodic mode (no knee parameter), the aperiodic component was modeled as L(f) = b—χ · log(f), where b is the broadband offset and χ the spectral exponent. The resulting aperiodic parameters (exponent and offset) were averaged across all valid channels (excluding channels where fitting failed, marked as NaN) to yield a single representative value per session. Periodic oscillatory parameters were extracted for identified peaks within theta (4–7 Hz), alpha (8–13 Hz), beta (14–30 Hz), and low‐gamma (31–40 Hz) bands. Goodness‐of‐fit was assessed using *R* ^2^. One session in the ID evening condition showed a marginally lower mean *R* ^2^ value (*R* ^2^ = 0.875); this session was retained to preserve the complete repeated‐measures design. All other sessions exceeded *R* ^2^ = 0.90.

### 2.8 Statistical Analysis

Statistical analyses were conducted using Python‐based toolkits (SciPy and Statsmodels). Two‐way mixed‐design ANOVAs (Group [ID vs. HC] × Time [evening vs. morning]) were performed for each parameter, with partial eta‐squared (ηp^2^) and 90% confidence intervals as effect sizes. Omnibus *p*‐values were corrected using the Benjamini–Hochberg FDR (*q* < 0.05), applied separately within each measurement domain (aperiodic, microstate temporal, and microstate transition parameters). Significant interactions were decomposed via planned simple effects (Welch's *t*‐tests for between‐group, paired *t*‐tests for within‐group comparisons; Cohen's d; uncorrected *p*‐values). Normality was assessed by Shapiro–Wilk tests; departures observed for several microstate variables were further evaluated using permutation tests with 5000 iterations, which yielded the same pattern of statistically significant and non‐significant effects as the parametric ANOVA results. Simple effects were treated as planned comparisons and reported without additional correction, consistent with standard practice for decomposing significant omnibus interactions.

To investigate the relationship between neural features and clinical outcomes, Spearman rank correlations with bootstrap 95% confidence intervals (5000 resamples) were computed within the ID group to examine associations between electrophysiological features showing significant ANOVA effects and clinical sleep indices (PSQI and ISI). An exploratory mediation analysis was performed based on the logic of Hayes' PROCESS macro. We tested an exploratory model in which microstate dynamics statistically mediated the association between the aperiodic exponent and sleep quality (Model: FOOOF Exponent → Microstate ms0 Mean Duration → PSQI). The significance of the indirect effect was assessed using bias‐corrected and accelerated (BCa) bootstrapping with 5000 resamples. Mediation type (full vs. partial) was determined by examining the significance of the direct effect after controlling for the mediator.

## 3 Results

### 3.1 Demographic and Clinical Characteristics

The ID group (*n* = 26) and HC group (*n* = 29) were well‐matched in demographic variables. No significant group differences were observed in age (ID: 49.04 ± 13.89 years vs. HC: 45.69 ± 11.29 years; *t* = 0.99, *p* = 0.330), sex distribution (χ2 = 0.00, *p* = 0.980), BMI (*t* = 0.25, *p* = 0.800), or years of education (*t* = −0.38, *p* = 0.710). As expected based on inclusion criteria, the ID group exhibited significantly higher PSQI scores (ID: 16.12 ± 3.62 vs. HC: 3.66 ± 1.17; *t* = 17.58, *p* < 0.001) and ISI scores (ID: 17.15 ± 4.07 vs. HC: 3.79 ± 1.66; *t* = 16.27, *p* < 0.001). No significant group differences were observed in global cognitive screening measures, including the MMSE (ID: 27.65 ± 1.88 vs. HC: 26.52 ± 3.09; *t* = 1.63, *p* = 0.110) or the MoCA (ID: 26.31 ± 5.24 vs. HC: 27.59 ± 1.35; *t* = −1.27, *p* = 0.210) (Table 1).

**TABLE 1:** Demographic and clinical characteristics of participants.

Characteristics demographics	ID group (*n* = 26)	HC group (*n* = 29)	Statistic value	*p*
Age (years)	49.04 ± 13.89	45.69 ± 11.29	*t* = 0.99	0.330
Sex (Male/Female)	8/18	9/20	*χ* ^2^ = 0.00	0.980
BMI (kg/m^2^)	24.50 ± 3.19	24.30 ± 2.71	*t* = 0.25	0.800
Education (years)	13.46 ± 4.08	13.83 ± 3.06	*t* = −0.38	0.710
Clinical assessments				
PSQI	16.12 ± 3.62	3.66 ± 1.17	*t* = 17.58	< 0.001*
ISI	17.15 ± 4.07	3.79 ± 1.66	*t* = 16.27	< 0.001*
MMSE	27.65 ± 1.88	26.52 ± 3.09	*t* = 1.63	0.110
MoCA	26.31 ± 5.24	27.59 ± 1.35	*t* = −1.27	0.210
Objective sleep metrics				
Sleep onset (hh:mm)	22:42 ± 60 min	22:54 ± 47 min	ICC = 0.96/0.77	—
Wake up time (hh:mm)	05:46 ± 55 min	06:16 ± 27 min	ICC = 0.95/0.89	—
Total sleep time (hours)	6.92 ± 1.20	7.23 ± 0.70	ICC = 0.83/0.74	—

*Note:* Data are presented as Mean ± SD or Count. ICC values are presented as ID/HC.

Abbreviations: BMI, Body Mass Index; HC, Healthy Controls; ICC, Intraclass Correlation Coefficient (Smartwatch vs. Sleep Diary); ID, Insomnia Disorder; ISI, Insomnia Severity Index; MMSE, Mini‐Mental State Examination; MoCA, Montreal Cognitive Assessment; PSQI, Pittsburgh Sleep Quality Index.

* *p* < 0.001; *p* < 0.01; *p* < 0.05.

Objective sleep metrics derived from Apple Watch monitoring corroborated the clinical phenotype. Total sleep time averaged 6.92 ± 1.20 h in the ID group and 7.23 ± 0.70 h in the HC group. Intraclass correlation analyses demonstrated moderate‐to‐high concordance between subjective sleep diaries and smartwatch‐derived measures (ID group ICCs: wake up time = 0.95, sleep onset = 0.96, total sleep time = 0.83; HC group ICCs: wake up time = 0.89, sleep onset = 0.77, total sleep time = 0.74), supporting the reliability of the sleep verification protocol (Table 1).

### 3.2 Microstate Topographies and Diurnal Dynamics

**FIGURE 1:**
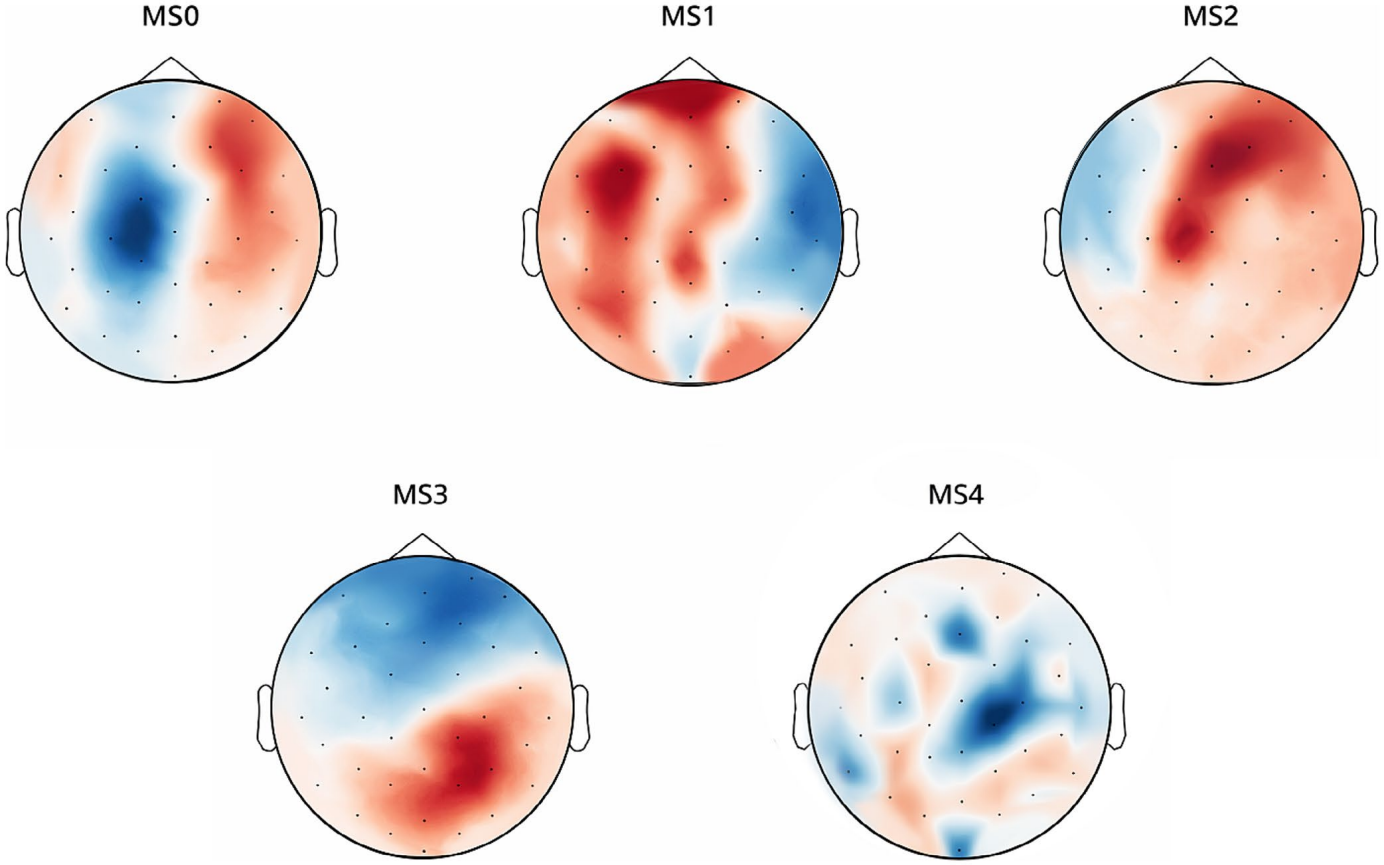
OPM‐MEG resting‐state microstate topographies. Cluster centroids (*k* = 5) identified via polarity‐invariant k‐means clustering, displayed on a flattened sensor array. OPM‐MEG, Optically Pumped Magnetometer‐Magnetoencephalography.

**FIGURE 2:**
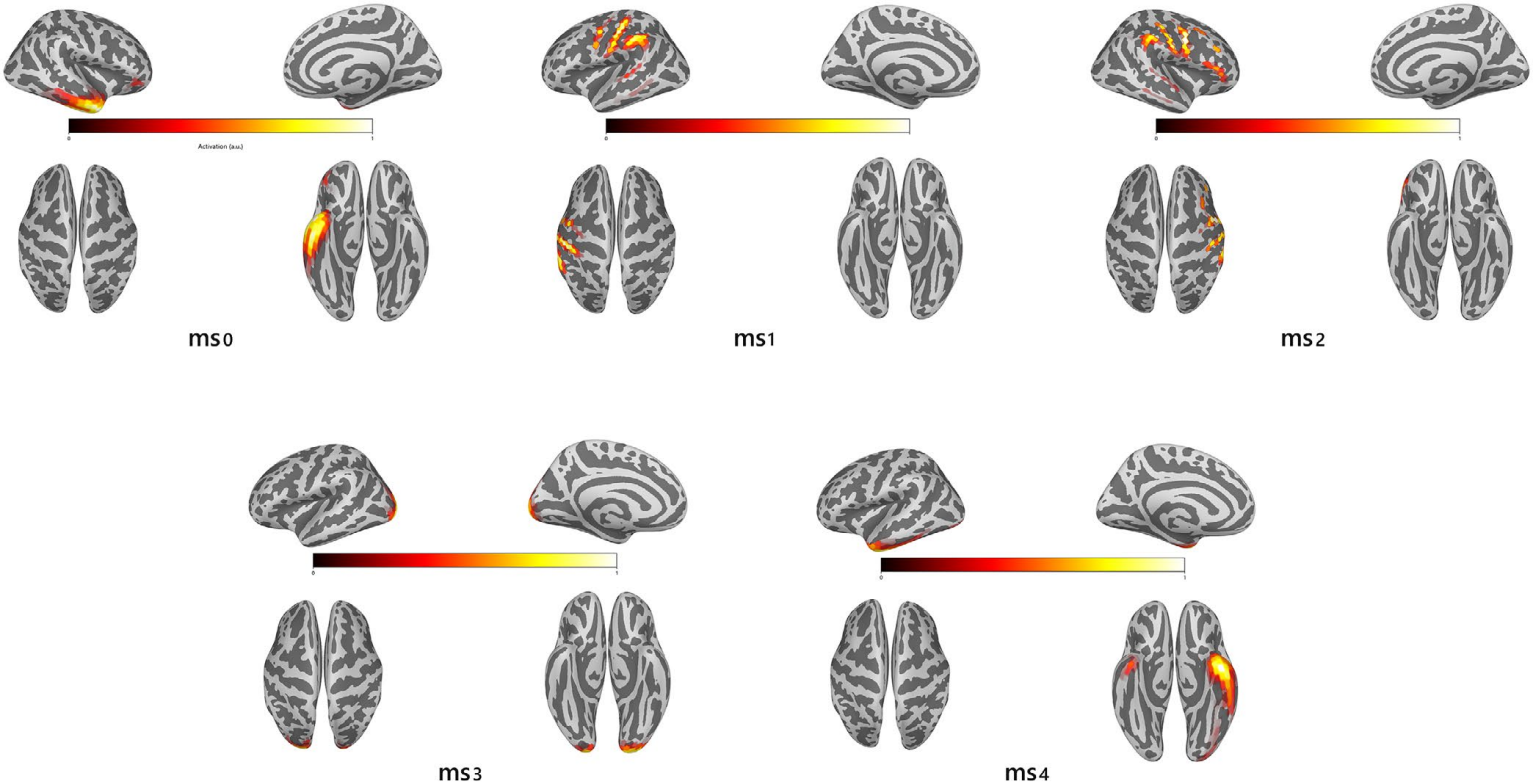
Cortical source reconstruction of the five microstate classes. Spatial distributions of ms0–ms4 were estimated using LCMV beamforming and mapped to Desikan–Killiany parcels. Colored regions indicate peak power distributions for each class. ms, microstate class; LCMV, linearly constrained minimum variance.

Group‐level clustering of OPM‐MEG resting‐state data identified five reproducible microstate classes (ms0–ms4) (Figure 1), collectively accounting for approximately 65% of the global field variance. Split‐half validation further supported the stability of this five‐class solution, including global template stability (Table S1) and per‐microstate stability (Table S2). Based on template‐based source reconstruction, source‐level projections revealed distinct cortical distributions associated with each microstate class, approximately mapping to a putative right temporal‐limbic network for ms0, a left sensorimotor and inferior parietal network for ms1, a right sensorimotor and fronto‐parietal network for ms2, a primary visual network for ms3, and a left temporal‐limbic network for ms4 (Figure 2).

A series of two‐way mixed‐design ANOVAs (Group × Time) with FDR correction applied within each measurement domain revealed significant interaction and main effects across multiple microstate parameters. For microstate ms0 mean duration, a significant Group × Time interaction was observed (*F* = 7.94, *p_FDR* = 0.034, ηp^2^ = 0.130, 90% CI [0.021, 0.270]). Simple effects analysis revealed that the ID group had significantly longer ms0 duration than controls in the evening (37.58 ± 2.50 vs. 36.13 ± 1.60 ms; Welch *t* = 2.53, *p* = 0.015, *d* = 0.70), whereas this pattern reversed in the morning (30.97 ± 20.96 vs. 53.06 ± 37.10 ms; *t* = −2.76, *p* = 0.008, *d* = −0.72). Within the HC group, ms0 duration was significantly higher in the morning than in the evening (*t* = −2.46, *p* = 0.020, *d* = −0.46), while the ID group showed no significant diurnal change (*p* = 0.141). A similar pattern was observed for ms0 coverage (*F* = 17.86, *p_FDR* = 0.001, ηp^2^ = 0.252, 90% CI [0.097, 0.394]): The ID group showed higher coverage in the evening (*t* = 3.74, *p* < 0.001, *d* = 1.05), and lower in the morning (*t* = −2.74, *p* = 0.009, *d* = −0.73). HC showed a significant morning elevation (*t* = −4.31, *p* < 0.001, *d* = −0.80); within‐ID diurnal change was not significant (*p* = 0.064). Microstate ms1 coverage exhibited both a significant Group main effect (*F* = 139.71, *p_FDR* < 0.001, ηp^2^ = 0.725, 90% CI [0.610, 0.788]) and a Group × Time interaction (F = 6.35, *p_FDR* = 0.044, ηp^2^ = 0.107, 90% CI [0.012, 0.243]). Simple effects confirmed that the ID group had persistently elevated ms1 coverage at both time points (evening: 30.2% ± 10.7% vs. 9.2% ± 5.5%, *t* = 9.00, *p* < 0.001, *d* = 2.51; morning: 25.3% ± 6.6% vs. 9.5% ± 3.1%, *t* = 11.15, *p* < 0.001, *d* = 3.12). Within the ID group, ms1 coverage decreased from evening to morning (*t* = 2.52, *p* = 0.018, *d* = 0.50), whereas HCs remained stable (*p* = 0.738). Additional significant Group × Time interactions were observed for ms2 coverage (*F* = 7.08, *p_FDR* = 0.039, ηp^2^ = 0.118, 90% CI [0.016, 0.256]) and ms2 occurrence (*F* = 8.02, *p_FDR* = 0.034, ηp^2^ = 0.131, 90% CI [0.022, 0.271]); simple effects indicated that these effects were driven by increased evening values in the ID group (ms2 occurrence: Evening ID 6.19 ± 3.00 vs. HC 4.49 ± 2.44, *t* = 2.29, *p* = 0.026, *d* = 0.63). For ms3, only a significant Time main effect was observed (*F* = 27.71, *p_FDR* < 0.001, ηp^2^ = 0.343), indicating normal diurnal variation in both groups without group‐specific disruption.

**FIGURE 3:**
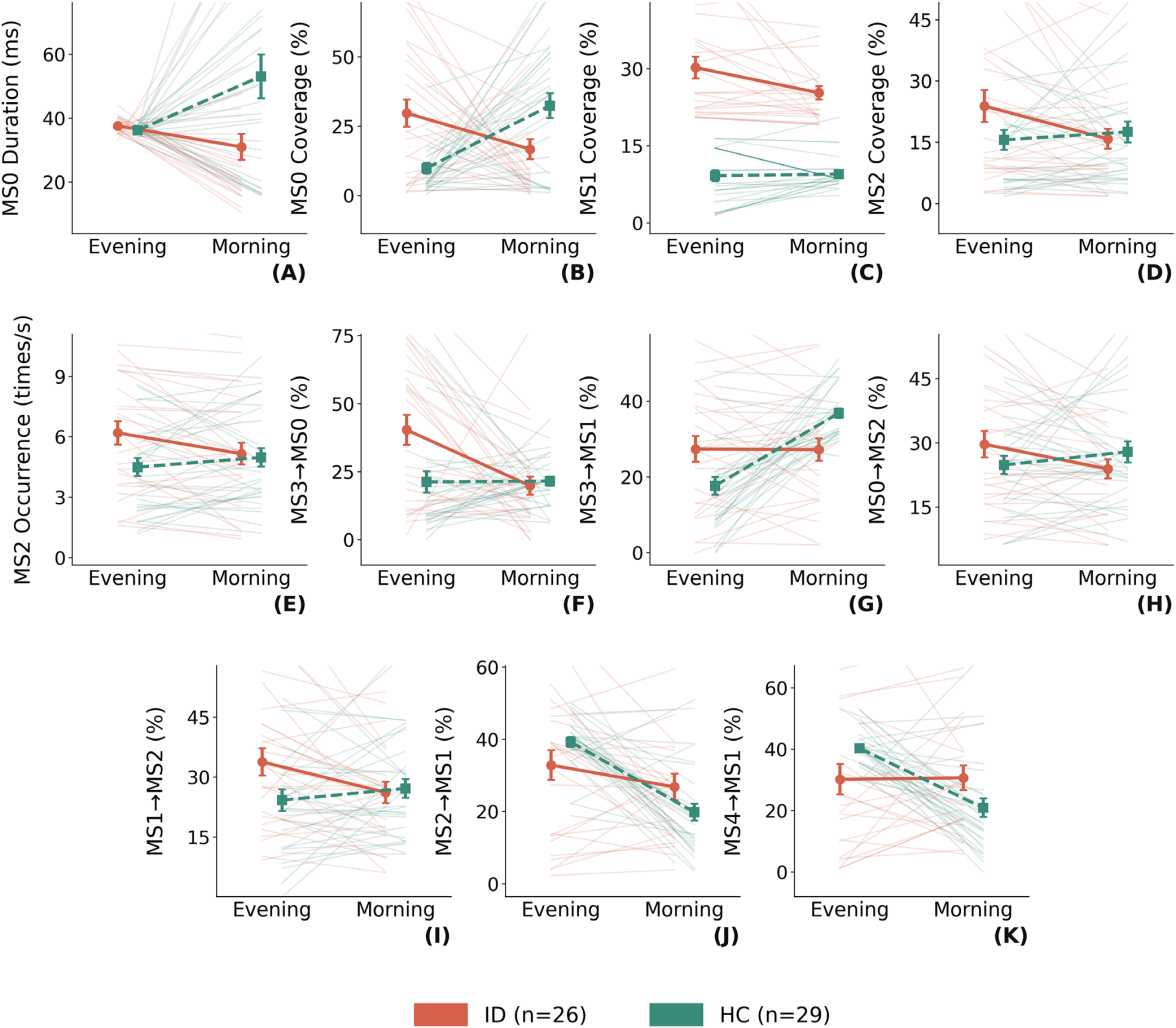
Group differences in microstate temporal dynamics and syntax. Individual trajectories (thin lines) and group mean ± SE (thick lines with error bars) are shown for ID (*n* = 26) and HC (*n* = 29) across Evening and Morning sessions. Annotations indicate significant two‐way mixed ANOVA effects (FDR‐corrected). *p_FDR < 0.05, **p_FDR < 0.01, ***p_FDR < 0.001. ID, Insomnia Disorder; HC, Healthy Controls.

Microstate transition analysis further revealed significant Group × Time interactions in multiple transition probabilities. Transitions from ms3 to ms0 showed a significant interaction (*F* = 7.14, *p_FDR* = 0.043, ηp^2^ = 0.119, 90% CI [0.016, 0.257]). Simple effects analysis revealed that the ID group exhibited significantly elevated ms3 → ms0 transitions in the evening (0.40 ± 0.28 vs. 0.21 ± 0.21; *t* = 2.84, *p* = 0.007, *d* = 0.78), with no group difference in the morning (*p* = 0.657). Within the ID group, ms3 → ms0 transitions increased significantly from morning to evening (*t* = 3.16, *p* = 0.004, *d* = 0.62), while HCs remained stable (*p* = 0.951). Transitions from ms3 to ms1 exhibited the strongest interaction (*F* = 25.41, *p_FDR* < 0.001, ηp^2^ = 0.324, 90% CI [0.156, 0.460]): The ID group showed higher evening transitions (0.27 ± 0.17 vs. 0.18 ± 0.13; *t* = 2.34, *p* = 0.024, *d* = 0.64), but lower morning transitions (0.27 ± 0.15 vs. 0.37 ± 0.07; *t* = −3.01, *p* = 0.005, *d* = −0.84). This crossover was driven by a marked within‐group decrease in HCs from morning to evening (*t* = −7.23, *p* < 0.001, *d* = −1.34), whereas the ID group showed no significant diurnal modulation (*p* = 0.952). Additional significant interactions were observed for ms0 → ms2 (*F* = 6.59, *p_FDR* = 0.044, ηp^2^ = 0.111, 90% CI [0.013, 0.247]; driven by increased evening transitions in ID, within‐ID: *p* = 0.010), ms1 → ms2 (*F* = 6.97, *p_FDR* = 0.043, ηp^2^ = 0.116, 90% CI [0.015, 0.254]; evening ID>HC, *t* = 2.20, *p* = 0.033), ms2 → ms1 (*F* = 7.82, *p_FDR* = 0.043, ηp^2^ = 0.129, 90% CI [0.021, 0.268]; driven by HC decrease from morning to evening, *p* < 0.001), and ms4 → ms1 (*F* = 13.54, *p_FDR* = 0.005, ηp^2^ = 0.203, 90% CI [0.063, 0.347]; HC morning‐to‐evening decrease, *p* < 0.001), collectively indicating widespread evening‐specific alterations in network switching dynamics in ID (Figure 3).

### 3.3 Aperiodic Spectral Components

**FIGURE 4:**
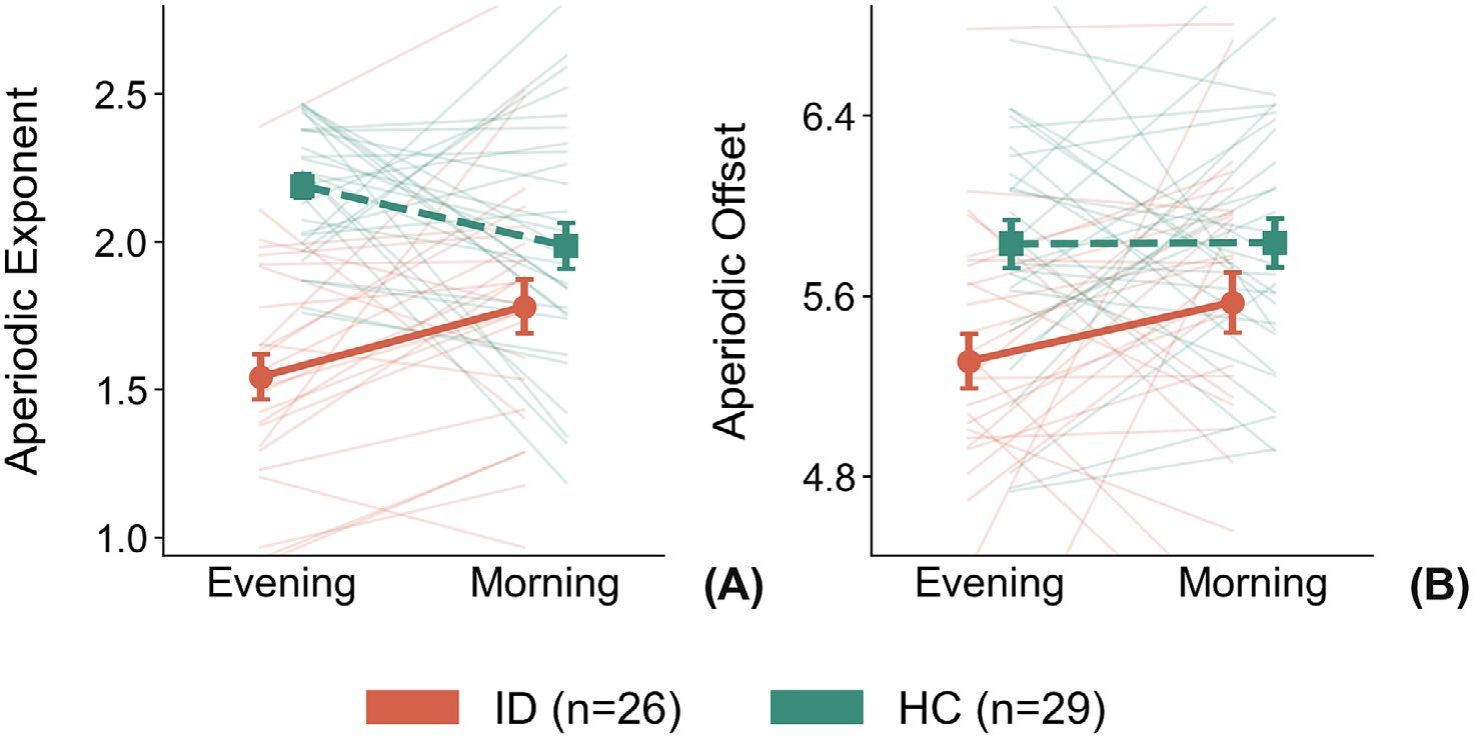
Aperiodic neural activity profiles and diurnal modulation. Individual trajectories (thin lines) and group mean ± SE (thick lines with error bars) are shown for the aperiodic exponent and offset across Evening and Morning sessions. Annotations indicate significant two‐way mixed ANOVA effects (FDR‐corrected). *p_FDR < 0.05, **p_FDR < 0.01, ***p_FDR < 0.001. ID, Insomnia Disorder; HC, Healthy Controls.

Neural power spectra were parameterized using the FOOOF algorithm to extract aperiodic components. Two‐way mixed ANOVA revealed that the aperiodic spectral exponent exhibited both a significant Group main effect (*F* = 25.57, *p_FDR* < 0.001, ηp^2^ = 0.325, 90% CI [0.157, 0.462]) and a significant Group × Time interaction (*F* = 13.44, *p_FDR* = 0.001, ηp^2^ = 0.202, 90% CI [0.062, 0.346]). Simple effects analysis showed that the ID group had a significantly lower exponent than controls in the evening (1.54 ± 0.39 vs. 2.19 ± 0.22; Welch *t* = −7.38, *p* < 0.001, *d* = −2.05), while the morning difference was not significant (1.78 ± 0.47 vs. 1.99 ± 0.42; *t* = −1.71, *p* = 0.093). Within the ID group, the exponent decreased significantly from morning to evening (*t* = −3.11, *p* = 0.005, *d* = −0.61), whereas HCs showed an opposite trend with the exponent increasing toward the evening (*t* = 2.24, *p* = 0.033, *d* = 0.42), indicating opposite diurnal trajectories of cortical excitability. The aperiodic offset showed a significant Group main effect only (*F* = 10.06, *p_FDR* = 0.003, ηp^2^ = 0.159, 90% CI [0.036, 0.302]): The ID group had lower offset values across both sessions (evening: 5.31 ± 0.62 vs. 5.83 ± 0.58; morning: 5.57 ± 0.68 vs. 5.84 ± 0.58), without significant diurnal modulation. No significant effects were detected for periodic oscillatory parameters (theta, alpha, beta, and low gamma) after correcting for the 1/f background (all *p* > 0.05) (Figure 4).

### 3.4 Brain‐Behavior Correlations

**FIGURE 5:**
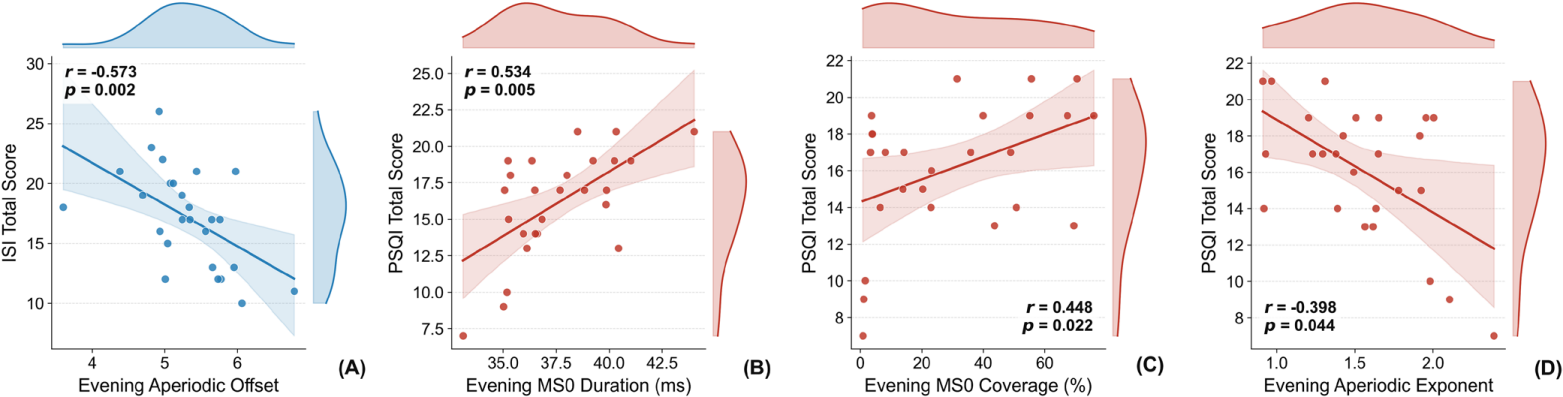
Correlations between electrophysiological features and sleep severity metrics. Scatterplots show Spearman rank correlations within the ID group (*n* = 26). Regression lines with bootstrap 95% CI bands are displayed. Marginal strips indicate kernel density distributions. Red, PSQI; Blue, ISI. PSQI, Pittsburgh Sleep Quality Index; ISI, Insomnia Severity Index. Electrophysiological features are consistently displayed on the x‐axis and sleep severity metrics on the y‐axis across all panels.

Spearman rank correlation analyses with bootstrap 95% confidence intervals were computed within the ID group for variables showing significant ANOVA effects. Four associations reached uncorrected *p* < 0.05. The evening aperiodic offset was negatively correlated with ISI (*r* = −0.573, *p* = 0.002, 95% CI [−0.827, −0.191]), indicating that lower broadband power was associated with greater insomnia severity. Evening ms0 mean duration was positively correlated with PSQI (*r* = 0.534, *p* = 0.005, 95% CI [0.093, 0.809]), indicating that longer limbic microstate stability was associated with poorer sleep quality. Evening ms0 coverage also correlated with PSQI (*r* = 0.448, *p* = 0.022, 95% CI [−0.006, 0.760]). The evening aperiodic exponent was negatively correlated with PSQI (*r* = −0.398, *p* = 0.044, 95% CI [−0.731, 0.048]), further linking elevated cortical excitability to subjective sleep disturbance (Figure 5). Of note, the bootstrap 95% CI for the latter two associations (ms0 coverage–PSQI and aperiodic exponent–PSQI) marginally crossed zero, warranting cautious interpretation. It should be noted, however, that the significance of the indirect effect in the subsequent mediation analysis is established via bootstrapping of the product of paths a and b, which constitutes a distinct and more sensitive test; the marginal crossing of zero in these bivariate correlations does not invalidate the mediation inference.

### 3.5 Mediation Analysis

**FIGURE 6:**
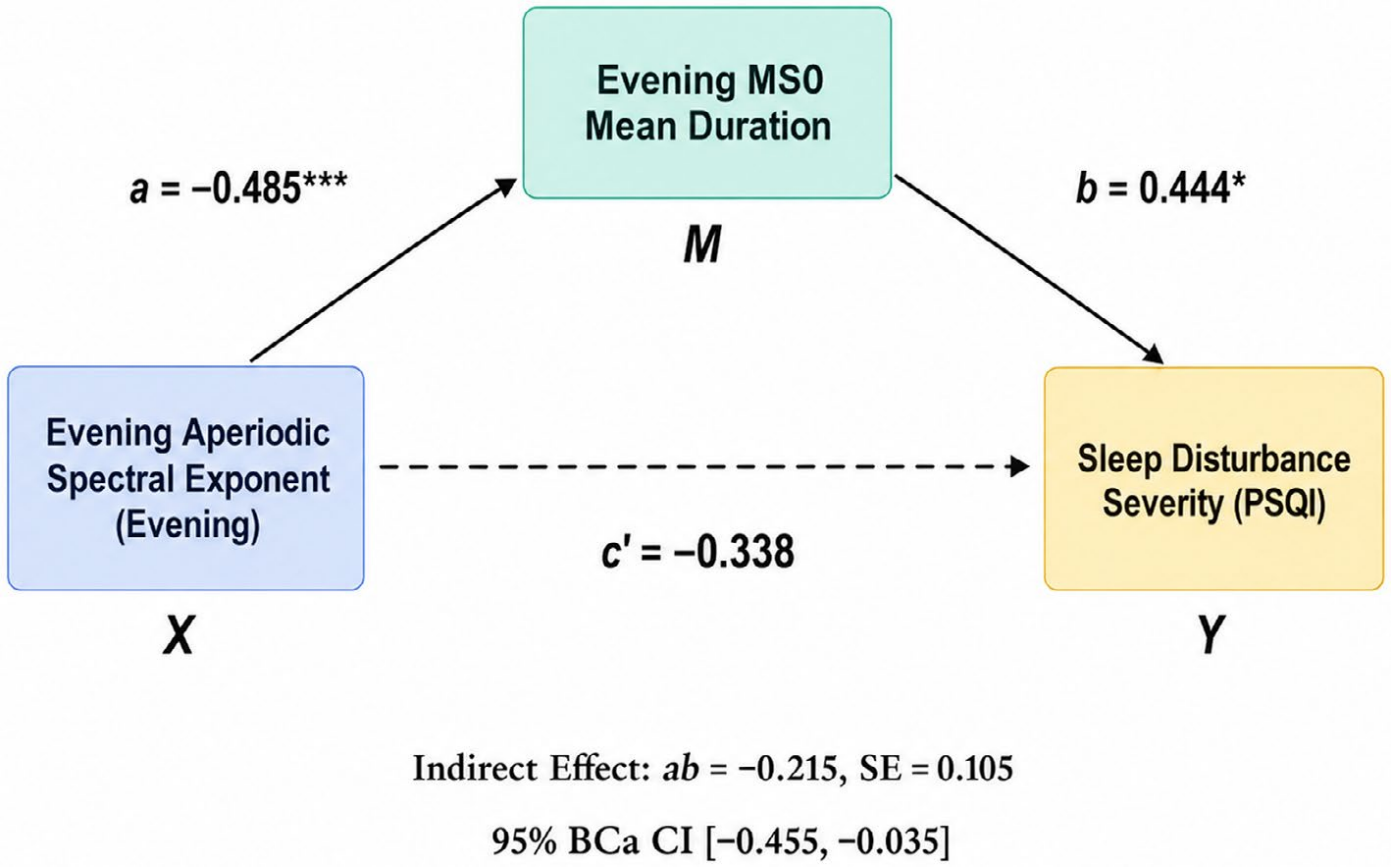
Mediation analysis of cortical excitability and sleep quality. Exploratory path diagram illustrating the indirect effect of the evening aperiodic exponent on PSQI scores through microstate 0 mean duration (ab = −0.215, SE = 0.105, 95% BCa CI [−0.455, −0.035]). Values represent standardized coefficients. Dashed line indicates a non‐significant path. Indirect effect tested via 5000 bootstrap resamples. *p < 0.05, **p < 0.001.

Mediation analysis within the ID group tested whether evening ms0 mean duration mediated the relationship between the aperiodic spectral exponent and subjective sleep quality. Using standardized coefficients, the evening spectral exponent significantly predicted ms0 duration (*a* = −0.485), and ms0 duration in turn predicted PSQI scores (*b* = 0.444). The indirect effect was significant (ab = −0.215, SE = 0.105, 95% BCa CI [−0.455, −0.035]), and the total effect (*c* = −0.553) was partially mediated, with a remaining direct effect of *c*′ = −0.338. The mediation proportion was 38.9%, indicating that approximately two‐fifths of the association between cortical excitability and sleep quality was accounted for by altered limbic microstate temporal dynamics (Figure 6).

## 4 Discussion

The present study employed wearable OPM‐MEG to investigate large‐scale brain network dynamics in ID across two critical time windows: The evening pre‐sleep period and the following morning. By jointly characterizing aperiodic spectral parameters and microstate dynamics, we identified a pattern of neural abnormalities that differed in both temporal specificity and network expression. Specifically, patients with ID exhibited evening‐specific alterations in aperiodic activity accompanied by abnormal stabilization of limbic‐related microstates, alongside a persistent dominance of sensorimotor network states present in both evening and morning recordings. This dissociation suggests that insomnia is characterized by the coexistence of highly evening‐specific dysregulations and more persistent, sustained disruptions in large‐scale brain dynamics [10, 23].

At the level of aperiodic activity, insomnia patients demonstrated a significantly flatter aperiodic spectral exponent and a reduced aperiodic offset during the evening compared with healthy controls. Prior work has established that the aperiodic exponent provides an indirect index of population‐level excitation–inhibition (E/I) balance, with flatter slopes reflecting a shift toward increased excitation and enhanced asynchronous neuronal firing [24, 38, 39]. It should be noted, however, that the link between the aperiodic exponent and E/I balance is considered indirect and context‐dependent, as the exponent may also reflect variations in neuronal firing rates, synaptic time constants, or other aspects of neural population dynamics [24, 38]. We therefore adopt a conservative interpretation, treating the observed exponent changes as a correlate of altered arousal‐related neural dynamics rather than a direct readout of synaptic excitation–inhibition ratio. Recent computational models further support that variations in this exponent track the transition between stable and chaotic network states, a critical factor for sleep onset [40]. Changes in the aperiodic offset are generally interpreted as reflecting alterations in baseline population firing activity, although their precise physiological determinants remain under investigation [24]. In the context of the present findings, the concomitant reduction of both exponent and offset in the evening suggests that patients with insomnia enter the pre‐sleep period with an abnormally elevated and poorly regulated background excitatory state. This pattern is consistent with the hyperarousal model proposing excessive cortical arousal prior to sleep onset [3, 7] and parallels the subjective experience of heightened alertness during the evening [12, 41]. Notably, the transition from wakefulness to drowsiness or early sleep is typically characterized by a steepening of the power spectrum (i.e., an increased aperiodic exponent). The observation of a significantly flattened exponent in the ID group during the evening session is therefore more consistent with a state of cortical hyperarousal than with drowsiness or early sleep‐stage contamination.

Importantly, the diurnal comparison further clarified the temporal specificity of this abnormality. While healthy controls exhibited stable aperiodic parameters across evening and morning sessions, insomnia patients showed significantly lower evening values relative to their own morning recordings. This finding indicates that the observed aperiodic alterations are not a fixed characteristic of the disorder but instead reflect a difficulty in appropriately reducing cortical excitability during the pre‐sleep period. Within frameworks of sleep homeostasis and arousal regulation, the evening is typically associated with a gradual reduction in cortical excitability in preparation for sleep [42, 43]. The persistence of a flattened aperiodic spectrum during this window suggests that this preparatory downregulation is disrupted in insomnia and may be related to altered thalamocortical gating and sensory disengagement processes necessary for sleep initiation [6, 44].

Against this altered excitability background, we observed pronounced changes in large‐scale network dynamics involving limbic‐related microstates. Microstate ms0, approximately mapped to putative temporal–limbic regions, exhibited significantly increased mean duration and global coverage in insomnia patients during the evening. At the same time, the visual‐related microstate ms3 showed a reduced mean duration in patients relative to controls during the evening session. These complementary effects suggest a redistribution of temporal resources away from sensory‐dominated states toward limbic configurations [9, 45]. From a dynamical systems perspective, transient brain states can be conceptualized as attractors in an energy landscape, with increased dwell time reflecting deeper or more stable attractor basins [11, 46]. Speculatively, elevated background excitability (flatter 1/f slope) may steepen these attractor basins for limbic‐related configurations, thereby reducing the flexibility of state transitions [47, 48]. This interpretation is further supported by microstate transition analyses. Insomnia patients exhibited an increased probability of transitions from ms3 to ms0 during the evening, indicating that visual or sensory‐related states are more likely to be followed by limbic network configurations. Rather than being effectively attenuated, incoming sensory information appears to be preferentially routed into emotion‐related processing streams. This dynamic pattern aligns with behavioral and neuroimaging evidence demonstrating persistent emotional processing, rumination, and slow disengagement from affective content in insomnia [49, 50].

In contrast to the highly evening‐specific limbic alterations, the sensorimotor‐related microstate ms1 exhibited a sustained elevation across both sessions. Notably, while ms1 coverage remained abnormally high in patients during both evening and morning relative to controls, it still exhibited significant diurnal modulation within the ID group, decreasing from evening to morning. Therefore, this persistent dominance of the putative sensorimotor network likely reflects an ongoing impairment in sensory gating that operates at an elevated set‐point across the sleep–wake boundary. Transition analysis revealed an increased probability of ms2 → ms1 transitions, suggesting that internally oriented or associative states more frequently give way to sensorimotor network dominance in patients. Functionally, suppression of sensorimotor activity during rest is thought to facilitate disengagement from bodily sensations [22]. Persistent dominance of this network may therefore reflect impaired sensory gating and heightened somatic monitoring, consistent with clinical phenomena such as sleep state misperception and increased bodily awareness in insomnia [51, 52]. The sensitivity of OPM‐MEG to sensorimotor rhythms likely facilitated the detection of this effect, which may be less apparent in hemodynamic imaging studies [27, 29].

The ANOVA framework revealed that insomnia‐related diurnal dynamics are not uniformly disrupted but rather show indicator‐specific profiles. The aperiodic exponent, ms0 temporal parameters (duration and coverage), and several transition probabilities exhibited significant Group × Time interactions, pointing to evening‐specific dysregulation in the ID group. In contrast, ms1 coverage displayed both a large Group main effect and a significant interaction, indicating a sustained elevation across both sessions with additional diurnal modulation. Together, these patterns suggest that insomnia involves indicator‐specific alterations in the temporal organization of brain dynamics, with limbic‐related network stabilization and cortical excitability changes concentrated at the sleep–wake boundary, while sensorimotor network dominance operates as a more persistent, time‐of‐day‐modulated phenomenon [43].

Mediation analysis provided an integrative perspective on the relationship between excitability, network dynamics, and subjective symptoms. Lower evening aperiodic exponent values were associated with increased limbic microstate ms0 mean duration, which in turn predicted poorer subjective sleep quality (PSQI). The indirect effect accounted for approximately 39% of the total association, indicating partial mediation. Both direct and indirect effects were present, indicating that limbic microstate dynamics partially mediate the relationship between cortical excitability and clinical severity [53]. These findings are consistent with a statistical framework in which altered background excitability is linked to large‐scale network dynamics associated with clinically relevant symptom variation. However, given the relatively small sample size within the ID group (*n* = 26) and the reliance on subjective questionnaire scores as the outcome measure, we emphasize that this mediation model is strictly exploratory. It serves as a statistical framework for linking physiological parameters to subjective experiences, rather than providing definitive mechanistic evidence [47].

Several limitations of the present study warrant acknowledgment. First, the absence of concurrent polysomnography constrains interpretation in two respects: Wakefulness during OPM‐MEG recordings was verified through video monitoring and verbal report rather than objective sleep staging, and fine‐grained sleep architecture parameters—including sleep stage proportions and micro‐arousal indices—could not be characterized, limiting direct linkage of electrophysiological findings to specific sleep architecture features. While the observed flattened aperiodic exponent in the ID group is inconsistent with drowsiness‐related spectral slowing, residual contamination cannot be fully excluded. Second, the absence of individual structural MRI necessitated template‐based source reconstruction, and microstate–anatomy mappings should therefore be interpreted as approximate and exploratory [37, 54, 55, 56, 57, 58, 59, 60]. Third, microstate analysis is sensitive to clustering parameters and model order selection, which may influence the resulting network assignments [13]. Although the *K* = 5 solution was selected using a data‐driven elbow criterion and repeated random initializations, future studies should further validate the robustness of this model order in larger independent cohorts. Fourth, the cross‐sectional design precludes causal inference, and the exploratory mediation model should be interpreted accordingly, underscoring the need for longitudinal and interventional follow‐up.

In summary, this study indicates that ID is characterized by temporally differentiated abnormalities in large‐scale brain dynamics. Evening‐specific dysregulation of cortical excitability was associated with altered limbic microstate stabilization and altered transition dynamics at the sleep–wake boundary, while persistent sensorimotor network dominance may reflect a sustained alteration in sensory gating. By leveraging the high temporal resolution of wearable OPM‐MEG [12, 28], these findings bridge background neural activity and large‐scale network organization, offering an electrophysiological framework for understanding hyperarousal and sleep initiation failure in ID.

## Author Contributions

Feng Cui contributed to the conceptualization and design of the study, methodology development, data acquisition, data curation, formal analysis, software implementation, visualization, interpretation of the results, and preparation of the original manuscript. Kejia Hu contributed to the conceptual development of the study, literature synthesis, interpretation of the findings, preparation of the original manuscript, and substantial revision of the manuscript. Hui Zeng, Yang Wang, and Xi Wang contributed to participant recruitment, clinical assessment, data acquisition, and data curation. Haiting Liu and Tangfen Wang contributed to participant recruitment and clinical coordination. Yan Chang, Xiaodong Yang, and Tao Hu provided technical support for OPM MEG data acquisition, system operation, and data quality control. Tianxiao Shen contributed to the clinical interpretation of the findings and critical revision of the manuscript. Jianbing Zhu contributed to supervision, project administration, clinical resource coordination, funding acquisition, and critical review and revision of the manuscript. Jian Zheng contributed to methodological supervision, technical coordination, project administration, funding acquisition, and critical review and revision of the manuscript. Donghua Pan contributed to study conceptualization, overall supervision, project administration, coordination among the participating teams, funding acquisition, and critical review and revision of the manuscript. All authors reviewed and approved the final version of the manuscript. Feng Cui and Kejia Hu contributed equally to this work and share first authorship.

## Funding

This work was supported by the Suzhou Science and Technology Innovation Project (SYG2025092); the Project of Suzhou Key Laboratory (SZS2025004); the Multi‐center Clinical Research on Major Diseases of Suzhou Municipal Health Commission (DZXYJ202525); the Suzhou Science and Technology Development Plan (2023SSD18); the Emergency Rescue, Research Foundation of Aviation Medicine (2025YJ009); the CAAE Epilepsy Research Fund (CS‐2025‐004); and the Suzhou Institute of Biomedical Engineering and Technology, Chinese Academy of Sciences (CX202601004).

## Ethics Statement

The study was conducted in accordance with the Declaration of Helsinki and was approved by the Ethics Committee of Suzhou Hospital, Affiliated Hospital of Medical School, Nanjing University (approval no. IRB202504008RI). The protocol was registered at the Chinese Clinical Trial Registry (registration no. ChiCTR2500111107). Written informed consent was obtained from all participants.

## Conflicts of Interest

Authors Yan Chang, Xiaodong Yang, and Tao Hu are employees of Suzhou Metanetic Co. Ltd., the manufacturer of the OPM MEG system used in this study. Their contribution was limited to technical support related to system operation and sensor calibration during data acquisition. The company and these authors were not involved in study design, participant recruitment, data analysis, statistical modeling, interpretation of results, or formulation of the manuscript conclusions. The remaining authors declare no competing interests.

## Supporting information


**Table S1:** Spatial correlations between half‐sample and global template maps across 50 random splits.

**Table S2:** Per‐microstate topography stability across all 100 half‐samples.

## Data Availability

The data that support the findings of this study are available from the corresponding author upon reasonable request.
