# Supplementary material for: Altered Evening Aperiodic Activity and Microstate Dynamics in Insomnia Disorder: An OPM MEG Study
Source: CNS Neurosci Ther. 2026 Jul 30;32(8):e71066. doi: 10.1002/cns.71066 (PMC13420205; doi:10.1002/cns.71066)
Supplement: Supplementary file 1 — Table S1: Spatial correlations between half‐sample and global template maps across 50 random splits. [file CNS-32-e71066-s002.docx]

**Supplementary Table S1. Spatial correlations between half-sample and global template maps across 50 random splits**

| **Split** | **Half A mean \|r\|** | **Half B mean \|r\|** | **Overall mean \|r\|** |
| --- | --- | --- | --- |
| 1 | 0.762 | 0.764 | 0.763 |
| 2 | 0.972 | 0.764 | 0.868 |
| 3 | 0.785 | 0.832 | 0.808 |
| 4 | 0.961 | 0.685 | 0.823 |
| 5 | 0.985 | 0.745 | 0.865 |
| 6 | 0.790 | 0.944 | 0.867 |
| 7 | 0.955 | 0.776 | 0.866 |
| 8 | 0.924 | 0.774 | 0.849 |
| 9 | 0.781 | 0.987 | 0.884 |
| 10 | 0.973 | 0.831 | 0.902 |
| 11 | 0.799 | 0.764 | 0.781 |
| 12 | 0.797 | 0.921 | 0.859 |
| 13 | 0.978 | 0.788 | 0.883 |
| 14 | 0.955 | 0.753 | 0.854 |
| 15 | 0.806 | 0.921 | 0.864 |
| 16 | 0.903 | 0.946 | 0.924 |
| 17 | 0.916 | 0.743 | 0.829 |
| 18 | 0.897 | 0.791 | 0.844 |
| 19 | 0.883 | 0.796 | 0.839 |
| 20 | 0.838 | 0.816 | 0.827 |
| 21 | 0.932 | 0.797 | 0.864 |
| 22 | 0.939 | 0.775 | 0.857 |
| 23 | 0.806 | 0.951 | 0.879 |
| 24 | 0.900 | 0.774 | 0.837 |
| 25 | 0.795 | 0.971 | 0.883 |
| 26 | 0.969 | 0.705 | 0.837 |
| 27 | 0.836 | 0.902 | 0.869 |
| 28 | 0.926 | 0.960 | 0.943 |
| 29 | 0.729 | 0.912 | 0.820 |
| 30 | 0.737 | 0.938 | 0.838 |
| 31 | 0.937 | 0.777 | 0.857 |
| 32 | 0.918 | 0.850 | 0.884 |
| 33 | 0.872 | 0.700 | 0.786 |
| 34 | 0.967 | 0.746 | 0.857 |
| 35 | 0.769 | 0.962 | 0.866 |
| 36 | 0.752 | 0.765 | 0.758 |
| 37 | 0.983 | 0.800 | 0.891 |
| 38 | 0.973 | 0.802 | 0.888 |
| 39 | 0.712 | 0.836 | 0.774 |
| 40 | 0.954 | 0.744 | 0.849 |
| 41 | 0.771 | 0.870 | 0.820 |
| 42 | 0.722 | 0.966 | 0.844 |
| 43 | 0.775 | 0.985 | 0.880 |
| 44 | 0.821 | 0.781 | 0.801 |
| 45 | 0.925 | 0.772 | 0.849 |
| 46 | 0.927 | 0.753 | 0.840 |
| 47 | 0.897 | 0.758 | 0.827 |
| 48 | 0.907 | 0.832 | 0.869 |
| 49 | 0.827 | 0.945 | 0.886 |
| 50 | 0.902 | 0.931 | 0.917 |
| **Mean ± SD** | **0.871 ± 0.083** | **0.832 ± 0.087** | **0.851 ± 0.039** |

**Note:** 55 participants were randomly divided into two halves (27 to 28 participants per half) at the subject level. GFP-peak topographies from each half were independently submitted to polarity-invariant modified k-means clustering (K = 5). The resulting half-sample template maps were matched to the global templates derived from all participants using the Hungarian algorithm with absolute spatial correlation. This procedure was repeated 50 times with different random partitions. Half A mean |r| and Half B mean |r| indicate the average absolute spatial correlation between each half-sample solution and the global template across the five microstate classes. Overall mean |r| indicates the average stability across both halves. Global GEV = 65.73%.
