# Supplementary material for: Altered Evening Aperiodic Activity and Microstate Dynamics in Insomnia Disorder: An OPM MEG Study
Source: CNS Neurosci Ther. 2026 Jul 30;32(8):e71066. doi: 10.1002/cns.71066 (PMC13420205; doi:10.1002/cns.71066)
Supplement: Supplementary file 2 — Table S2: Per‐microstate topography stability across all 100 half‐samples. [file CNS-32-e71066-s001.docx]

**Supplementary Table S2. Per-microstate topography stability across all 100 half-samples**

| **Microstate** | **Mean \|r\| ± SD** |
| --- | --- |
| MS0 | 0.851 ± 0.234 |
| MS1 | 0.853 ± 0.227 |
| MS2 | 0.802 ± 0.262 |
| MS3 | 0.857 ± 0.216 |
| MS4 | 0.893 ± 0.166 |

**Note:** Values represent the mean absolute spatial correlations for each microstate class across all 100 half-samples generated from 50 random split-half validations. In each split, 55 participants were randomly divided into two halves (27 to 28 participants per half) at the subject level. GFP-peak topographies from each half were independently submitted to polarity-invariant modified k-means clustering (K = 5). The resulting template maps were matched to the global templates derived from all participants using the Hungarian algorithm with absolute spatial correlation. Global GEV = 65.73%.
